# Contamination of Wheat Cultivated in Various Regions of Poland during 2017 and 2018 Agricultural Seasons with Selected Trichothecenes and Their Modified Forms

**DOI:** 10.3390/toxins11020088

**Published:** 2019-02-01

**Authors:** Marcin Bryła, Edyta Ksieniewicz-Woźniak, Tomoya Yoshinari, Agnieszka Waśkiewicz, Krystyna Szymczyk

**Affiliations:** 1 Department of Food Analysis, Prof. Waclaw Dabrowski Institute of Agricultural and Food Biotechnology, Rakowiecka 36, 02-532 Warsaw, Poland; edyta.wozniak@ibprs.pl (E.K.-W.); krystyna.szymczyk@ibprs.pl (K.S.); 2 Division of Microbiology, National Institute of Health Sciences, 3-25-26 Tonomachi, Kawasaki-ku, Kawasaki-shi, Kanagawa 210-9501, Japan; t-yoshinari@nihs.go.jp; 3 Department of Chemistry, Poznan University of Life Sciences, Wojska Polskiego 75, 60-625 Poznan, Poland; agnieszka.waskiewicz@up.poznan.pl

**Keywords:** trichothecenes, nivalenol-3-glucoside, deoxynivalenol-3-glucoside, wheat, weather conditions

## Abstract

Cross-interaction of antibodies within the immunoaffinity columns used in this study facilitated the simultaneous determination of nivalenol (NIV), deoxynivalenol (DON), their glucoside derivatives (NIV-3G, DON-3G), and 3-acetyl-deoxynivalenol (3-AcDON) in wheat grain harvested in various regions of Poland. In Poland, 2018 was a warm, dry agricultural season, and hence, was relatively less favourable for cereal cultivation than 2017. Data on the natural occurrence of NIV-3G in wheat grain are among the first published in the literature. DON was the most frequently found mycotoxin in the tested samples; the percentage occurrence of DON-positive samples was 92% in 2017 and 61% in 2018. Moreover, DON concentrations were generally higher in 2017 samples (5.2–1670.7 µg/kg) than those in 2018 samples (range 5.0–461.7 µg/kg). A similar pattern was found for DON-3G. However, no statistically significant differences between the samples from the two agricultural seasons were observed for the other three mycotoxins that were analysed, and their concentrations were generally considerably lower. DON was strongly correlated with DON-3G (correlation coefficient *r* = 0.9558), while NIV was strongly correlated with NIV-3G (*r* = 0.9442). The percentage occurrence of NIV-3G- and DON-3G-positive samples was 14% in 2017 and 49% in 2018. The NIV-3G/NIV ratio was 5.9–35.7%, while the DON-3G/DON ratio range was 3.2–53.6%. In 2018, wheat samples from Southern Poland exhibited statistically significantly higher levels of DON than those from Northern Poland. The dry and hot summer of 2018 not only reduced wheat yields, but also limited development of *Fusarium* spp. Therefore, grain harvested that year was generally contaminated with relatively low levels of mycotoxins. Lower levels of DON were also accompanied by lesser amounts of DON-derivatives.

## 1. Introduction

*Fusarium* head blight (FHB) is a dangerous cereal disease caused by the *Fusarium* fungi, in particular *F. graminearum* and *F. culmorum*. Infection of cereal plants accounts for significant losses in cereal crops all over the world. Pathogens responsible for the disease biosynthesize secondary metabolites commonly referred to as mycotoxins [1,2]. Infection of plants with the fungi, and consequent contamination of crops with mycotoxins, may be facilitated by environmental stress experienced by plants during their growth, particularly at the ear formation stage [3]. The fungi may change their metabolism in reaction to conditions, both natural and agrotechnological, prevailing in the plants’ cultivation environment [4].

The four types of toxins produced by the fungi are referred to as trichothecenes A, B, C, and D. The most important of these, from the food safety point of view, are type A trichothecenes (including HT-2 and T-2 toxins) and type B trichothecenes (including deoxynivalenol (DON), nivalenol (NIV), 3-acetyl-deoxynivalenol (3-AcDON), and 15-acetyl-deoxynivalenol (15-AcDON)) [5,6]. The compounds are most often found in wheat, barley, oats, and maize grain [7]. 

It is a well-established that weather conditions are one of the major factors determining the occurrence and extent of fungal infections [8,9,10,11]. The most crucial conditions include air temperature [12,13,14], rainfall [14], and air humidity [13,14]. Different specifies of fungi belonging to the *Fusarium* family may dominate in various climatic zones; for example, *F. graminearum* prefer warmer zones, while *F. culmorum* prefer colder regions [15,16]. Moreover, they may depend on the cultivated wheat variety [17]. 

Unfavourable effects of exposure to the compounds include nausea and vomiting, diarrhoea, and gastro-enteritis. Since crops are also major components of feed used in livestock production, trichothecenes in grain contribute to animal weight-loss, making them an economic liability [18]. For all these reasons, maximum permissible levels (MPLs) by law for certain mycotoxins in some cereal foodstuffs have been set out in numerous countries. In the European Union, DON MPLs range from 200 µg/kg in processed food for children, to 1750 µg/kg in unprocessed maize and durum wheat grain [19]. In animal organisms, NIV is more toxic than DON: the LD_50_ doses in mice amounted to 78 and 39 mg/kg for DON and NIV, respectively [20]. Conversely, DON is more toxic in plants than NIV [21,22]. 

In 2010, the Committee of FAO/WHO Experts on Food Additives decided that regulations limiting DON provisional maximum tolerable daily intake (PMTDI) should also include DON acetyl-derivatives. This decision reflected the observation that the latter compounds may undergo de-acetylation processes in the human gastrointestinal tract, and therefore can potentially threaten human health in the same manner as DON. Literature data from both in vitro and in vivo studies in animals have shown that deoxynivalenol-3-glucoside (DON-3G) is less toxic; however, intestinal bacteria residing in the lower part of the alimentary tract may hydrolyse DON-3G, and thereby also threaten human health [23,24,25,26]. The combined PMTDI for DON, DON acetyl-derivatives, and DON-3G was set by the Committee at 1.0 µg per kg body weight per day [27].

Using FHB-resistant wheat cultivars may be a crucial strategy to restrict occurrence of mycotoxins in cereal grains [28]. Resistance to FHB depends on plant height, blossoming time, lodging resistance, etc. [29], and is a quantitative and wildly varying feature, controlled by numerous gene loci [30]. It is frequently classified into five types: resistance to primary infection (type I), resistance to pathogen spreading (type II), resistance to grain infection (type III), tolerance to infections (type IV), and plant resistance to toxins (type V). The latter resistance type is based on two mechanisms: (i) metabolic transformation of DON to less-toxic compounds via glycosylation reactions; (ii) inhibition of trichothecene biosynthesis [31]. The so-called “modified mycotoxins” related to type V resistance have been the topic of numerous recent studies by scientists all over the world and have been identified due to advances in the development of analytical chemistry methods [32,33]. The ability of a plant to modify mycotoxins depends on the source of its resistance [30].

Knowledge of mutual relations between mycotoxins and their glucosides in cereal grains is important to correctly assess food safety. The aim of this study was thus twofold: (i) to develop an analytical method to simultaneously determine NIV, DON, their glucosides, and 3/15-AcDON in wheat grain samples, and (ii) to relate the contamination (with the aforementioned compounds) of wheat grain harvested in various regions of Poland in 2017 and 2018 to weather conditions prevailing in the regions during the respective wheat agricultural seasons. 

## 2. Results and Discussion

### 2.1. Method Validation

Linearity ranges, limits of detection (LOD, a concentration at which signal: Noise ratio was 3), limits of quantification (LOQ, a concentration at which signal: Noise ratio was 10), recovery rates (R%), and repeatability and precision (expressed as relative standard deviation RSD% in calibration curves) were determined from blank samples fortified with various amounts of NIV, DON-3G, or DON by means of subsequent dilutions of the standards. Calibration curves were measured in the following ranges [in µg/kg]: 8.0–560.2 for NIV; 5.0–480.2 for NIV-3G; 5.0–580.6 for DON; 4.0–85.3 for DON-3G; and 2.0–590.2 for 3/15-AcDON. Determination coefficients (R^2^) were high: 0.9909, 0.9905, 0.9891, 0.9910, 0.9974, 0.9901 for NIV, NIV-3G, DON, DON-3G, and 3/15-AcDON, respectively. LODs were equal to 8, 5, 5, 4, 2, and 2 µg/kg, respectively, while LOQ were equal to 24, 17, 17, 13, 7, and 7 µg/kg, respectively. Repeatability and recovery rates determined for four selected fortification levels are listed in Table 1. The lower-than-10% recovery rate for 15-AcDON was considered unsatisfactory. Apart from that single result, recovery rates for other analysed mycotoxins were within the 69.5–104.3% range, depending on the analyte and fortification level. RSD ranged from 4.5 to 20.2%.

Analytical methods used in EU to determine mycotoxins currently need to meet requirements specified within EC Regulation No. 401/2006 [34]. Method recovery and precision (relative standard deviations within data sets of results of repeated analyses) have been specified in that Regulation only for DON but not for the four other mycotoxins discussed in this paper: the former should be within the 60–120% range depending on the fortification level, the latter must not be worse than 20%. Taking into account that mycotoxins analysed in this paper are either DON-derivatives (DON-3G, 3-AcDON) or at least jointly with DON belong to the trichothecenes group (NIV, NIV-3G), the requirements specified for DON were used to evaluate performance of the method in determination of all five studied mycotoxins. Taking such an approach, it can be concluded that method validation results were satisfactory for each of them.

Cross-interaction of antibodies within the immunoaffinity columns used in this study made it possible to determine in wheat grain samples, not only NIV and DON, but also their glucoside derivatives and 3-AcDON. Unfortunately, the antibodies did not interact with 15-AcDON, and therefore recovery rates for that compound were below 10%, regardless of the fortification level. The cross-interaction of IAC column antibodies that help to determine derivatives of mycotoxin in cereal grains was previously employed by Yoshinari et al. [35], Geng et al. [36], Trombete et al. [37], and Bryła et al. [38]. However, to our knowledge, ours is the first report that the scope of substances that can be simultaneously determined in cereal grains has been extended by the use of IAC columns to include NIV, DON, DON-3G, NIV-3G, and 3-AcDON. 

### 2.2. NIV, DON, Their Glucosides, and 3-AcDON in Wheat Grain

The mild and temperate climate in Poland is mostly determined by air masses flowing in from various directions. That factor may influence weather conditions differently each year, and the susceptibility of wheat plants to the *Fusarium* fungal infection largely depends on these varying conditions. Different regions of the country have concomitantly varying landscapes and different distances to the Baltic sea, which may also influence the conditions. Therefore, information on agricultural season and wheat cultivation location were retained for all the analysed samples. 

Of a total 300 wheat grain samples, 150 were sampled in 2017, and 150 in 2018. DON, NIV, and DON-3G were the most abundant among all successfully determined mycotoxins (NIV, NIV-3G, DON, DON-3G, 3-AcDON): they were found in 92, 49, and 65% of all 2017 samples, and in 61, 59, and 33% of all 2018 samples, respectively. NIV-3G and 3-AcDON were found in 15 and 25% of all 2017 samples and in 13 and 16% of all 2018 samples, respectively. Complete results, broken down into both agricultural seasons and the five regions of Poland under consideration, are given in Table 2. The EU has regulated the maximum permissible level of DON in unprocessed cereal grains (except maize) to be 1250 µg/kg [19]. In the majority of the tested samples, the concentration of DON was far below that threshold. However, although the threshold was not exceeded in any of the 2018 samples, it was exceeded in 3 samples harvested in 2017. The 2017 samples contained more DON (average 109.2 µg/kg, range 5.2–1670.7 µg/kg) than those from 2018 (average 32.4 µg/kg, range 5.0–461.7 µg/kg). Consequently, 2017 samples contained also more DON-3G (average 25.8 µg/kg, range 4.0–217.2 µg/kg) than those from 2018 (average 12.3 µg/kg, range 4.0–87.7 µg/kg). Average concentrations of the three other tested mycotoxins did not differ statistically between 2017 and 2018. No statistically significant differences were revealed between the DON concentration in the 2017 samples from various regions of Poland. However, such differences were revealed for NIV and 3-AcDON. The NIV concentration in samples from Eastern Poland (average 14.6 µg/kg, range 8.1–34.3 µg/kg) was statistically different than that present in samples from Southern Poland (average 31.5 µg/kg, range 8.5–185.6 µg/kg). The 3-AcDON concentration in samples from Western Poland (average 5.7 µg/kg, range 2.5–8.9 µg/kg) was statistically different than that present in samples from Northern Poland (average 3.2 µg/kg, range 2.3–4.0 µg/kg), Central Poland (average 3.6 µg/kg, range 2.4–6.8 µg/kg), and Eastern Poland (average 2.7 µg/kg, range 2.0–3.4 µg/kg).

The percentage of the 2018 samples contaminated with mycotoxins was generally lower than that of the 2017 samples, e.g., 35–69% of 2018 samples were contaminated with DON, as compared to 53–100% of 2017 samples (varying by the cultivation region). Contamination with DON was higher in Southern Poland (average 55.1 µg/kg, range 5.0–461.7 µg/kg) than in Northern Poland (average 11.4 µg/kg, range 5.1–29.7 µg/kg). Even if the percentages of Western, Eastern, and Southern Poland samples contaminated with NIV (59, 79, and 68%, respectively) were higher than those of samples contaminated with DON (35, 67, and 42%, respectively), concentrations of NIV in Eastern and Southern Poland (average 22.6 µg/kg, range 8.2–74.5 µg/kg and average 19.7 µg/kg, range 8.0–61.9 µg/kg, respectively) were lower than the respective concentrations of DON (average 30.2 µg/kg, range 5.0–303.1 µg/kg and average 55.1 µg/kg, range 5.0–461.7 µg/kg, respectively).

**Figure 1:**
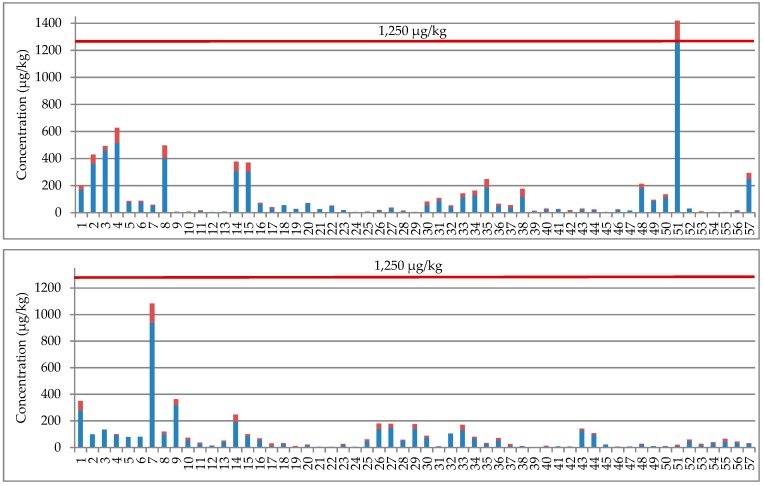

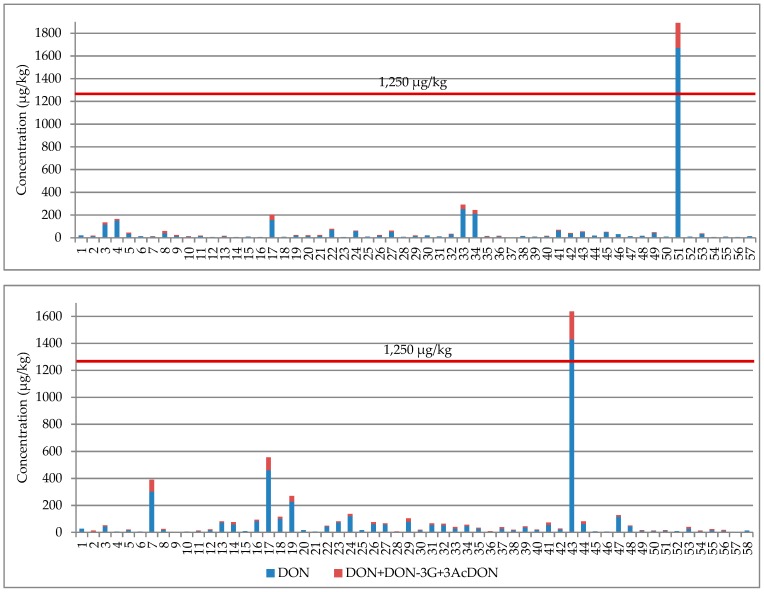
Concentrations of DON and total concentrations of DON+ DON-3G+3-AcDON in DON-positive wheat samples (*n* = 229). Maximum acceptable DON level in wheat grain (1250 μg/kg) specified in EC Regulation No. 1881/2006 [19] is shown for reference.

As a consequence of DON derivatives, EU-specified maximum acceptable DON level in wheat (1250 µg/kg) might be exceeded even if the found concentrations of DON alone were below that threshold. For that reason, total concentrations of DON+DON-3G+3-AcDON have been shown in Figure 1 for all DON-positive samples. It should be pointed out that DON legal threshold was not exceeded by the above specified total concentrations in any of the samples, except for the three samples containing DON itself above that threshold. Relatively low DON levels found in the tested samples suggest relatively low risk from the food safety point of view. However, one must always remember that the risk may be potentially increased by DON derivatives. 

**Figure 2:**
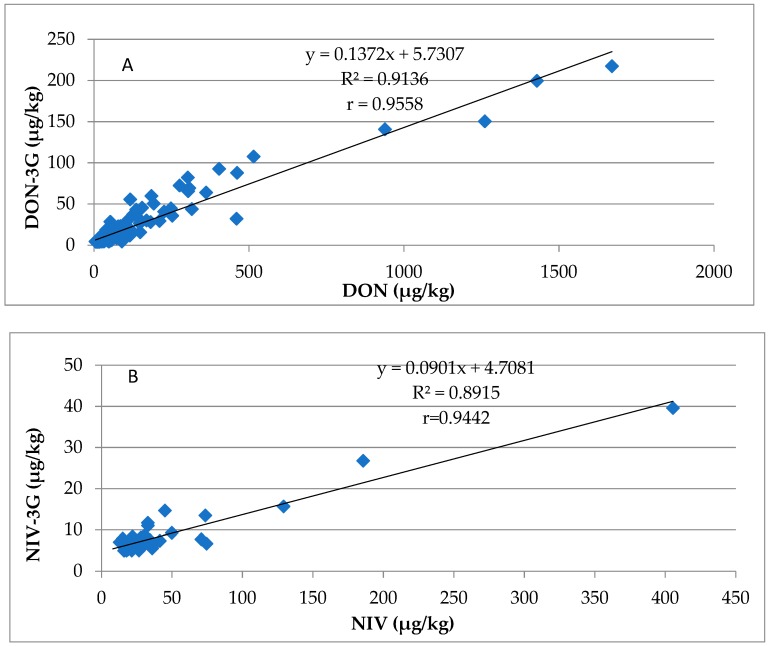
Correlation between concentrations of DON/DON-3G (**A**) and NIV/NIV-3G (**B**), ▪: wheat samples.

Concentrations of NIV/DON glucosides in wheat grain depend on the ability of wheat plants to biologically transform NIV/DON mycotoxins into their derivatives (second phase detoxication). Of the total 2017 and 2018 samples, 14% and 49% were contaminated with NIV-3G and DON-3G, respectively. Basic analogues were found in each such sample at higher or much higher levels than the levels of their glucosides. The molar ratios of NIV-3G/NIV and DON-3G/DON were 5.9–35.7% and 3.2–53.6%, respectively. Correlation between the concentration of DON/DON-3G and that of NIV/NIV-3G is shown in Figure 2 (top and bottom, respectively). The correlation coefficients, *r* = 0.9558 and 0.9442, respectively, reveal a very strong correlation. 

Food safety considerations suggest taking into account in any routine analysis of mycotoxins not only DON, but also DON-3G, even if EU regulations specify only DON maximum acceptable level in wheat grain. Because of strong DON/DON-3G correlation, concentrations of the latter might be evaluated on the basis of some determined concentration of the former. Our results show that 143 µg/kg of DON-3G may be expected for each 1000 µg/kg of DON found in wheat grain. In view of the above, the debate on whether to decrease the legally binding DON threshold from 1250 µg/kg down to (for example) 1000 µg/kg or not seems to be legitimate. However, the debate must be based, as much as possible, on analytical results on occurrence of mycotoxins in question, not only in wheat grain but also in other corn species. 

Weather conditions in 2018 were extremely unfavourable for cereal cultivation in most EU countries, including Poland. The volume of 2018 wheat crops in Poland is estimated at 9.9 million tons, i.e., 1.8 million tons less than was harvested in 2017. The dramatic drop can be attributed to much lower precipitation and higher temperatures prevailing in Poland during the 2018 agricultural season than were experienced in 2017. According to data published by the National Institute of Meteorology and Water Management in Warsaw, monthly rainfall in Poland during the wheat growth period in 2017 versus 2018 was: 64.3 versus 26.9 mm in April; 46.1 versus 44.3 mm in May; 75.8 versus 52.0 mm in June; 101.1 versus 94.1 mm in July; and 71.3 versus 40.0 mm in August. The average temperatures for respective months of 2017 versus 2018 were: 7.1 versus 12.8 °C; 13.6 versus 16.6 °C; 17.6 versus 18.2 °C; 18.1 versus 19.9 °C; and 19.0 versus 20.4 °C. As can be seen, weather conditions in 2018 were tough for cereal plant growth (water deficit, relatively high temperatures), and influenced crops quite heavily. The *Fusarium* fungi infect plants during dry and hot weather much less readily than during humid and colder weather [22]. This may be seen also in our results—contamination of our 2018 samples with mycotoxins was clearly lower than the contamination of the 2017 samples. 

However, some authors believe that hot and dry weather during the ear formation stage facilitates infection of plant bases with *F. graminearum* and *F. culmorum*; as soon as later storms bring abundant rainfall, the fungi may easily spread all over entire plants [39]. Abundant rainfall during the ear formation and blossoming stages as a factor heavily supporting the development of FHB is noted by numerous authors [40,41]. Lengthy wide-area rainfall was practically absent during the entire 2018 agricultural season in Poland; the majority of rainfall was brought about by short-lived local storms developing at relatively high air temperatures. Therefore, weather conditions can be said to have varied wildly from one region of the country to another.

NIV was found in a somewhat larger percentage of our 2018 samples (40–79%) than in the 2017 samples (18–71%), depending on the cultivation region. Average NIV concentrations were similar across the 2017 and 2018 samples, but the maximum level found in the 2018 samples was 405.4 µg/kg, whereas that found in the 2017 samples was only 185.6 µg/kg. This result is in line with report in the literature that biosynthesis of NIV by wheat-infecting fungi is more effective in warm environments than in colder environments. This is the converse of the effectiveness of biosynthesis of DON [42]. However, it is important to note that NIV absolute levels in cereal grains are generally much lower than DON levels [31,43].

Our results on DON/NIV in wheat correspond with data published by other European researchers. DON has been frequently found in European wheat grain, while the percentage of NIV-positive samples and NIV concentration levels is clearly lower. Several published results on DON and NIV in wheat grain give the following figures: Italy—28% (16/57) (9.6–99.6 µg/kg) [44]; 62.8% (27/43) (13–1230 µg/kg) [45]; Hungary—78.2% (287/367) (70–1560 µg/kg) [46]; Germany—100% (19/19) (15–1379 µg/kg) [47]); United Kingdom—86% (1396/1624) (max. 20333 µg/kg) [48]; Czech Republic—100% (48/48) (17.0–2265.2 µg/kg) [49], Croatia—65% (33/51) (max. 278 µg/kg) [50]; and Switzerland—80% (548/686) (max. 10 600 µg/kg) [32]. The frequency and the level of occurrence of NIV in wheat grain were much lower than that of DON: Italy—19.3% (11/57) (12-106 µg/kg) [44]; Hungary—9% (33/367) (50–590 µg/kg) [46]; Germany—26% (5/19) (max. 25 µg/kg) [47]; United Kingdom—67% (1088/1624) (max. 430 µg/kg) [48]; Czech Republic—78% (32/41) (15.4–25.9 µg/kg) [49]; and Switzerland—21% (144/686) (max. 470 µg/kg) [32].

Differences between DON concentrations in our 2017 samples originating from various different regions of Poland were statistically insignificant. However, the percentage of positive samples was higher in grain originating in Northern, Central, and Western Poland (100, 97, and 94%, respectively), than in Southern and Eastern Poland (53 and 79%, respectively). As can be seen in Figure S1 (Supplementary Materials) (breakdown of weather conditions by agricultural month and by the 16 regions of Poland studied), in most regions of Northern, Central, and Western Poland, total rainfall was higher in 2017 than in 2018 (and it was distributed somewhat more evenly throughout the agricultural season), while average temperatures were lower in 2017 than in 2018 by several degrees. However, total rainfall in 2018 was generally higher in Southern Poland, which can be correlated with the fact that the average DON concentration in 2018 was statistically significantly higher in samples from Southern Poland than in samples from Northern Poland.

Species dominating in a given region may also vary depending on weather conditions prevailing in a given season [16,17]; in fact, an observation that the degree to which any given plant variety cultivated at any given region is susceptible to FHB infection (and is consequently contaminated with mycotoxins) is dependent on prevalent weather conditions within that region during the given agricultural season is among the major conclusions of a 20-year-long project to monitor mycotoxin levels in cereal grains conducted in Finland, Sweden, Norway, and the Netherlands. This study showed that 46% of all n = 6,382 studied samples, most of which were wheat, maize, and oat samples, were DON-positive. A breakdown of the percentages of DON-positive wheat samples (and the maximum DON concentrations found, in µg/kg) by the countries covered is as follows: 20.6% = 114/554 samples (max. 890 µg/kg) in Sweden; 29.4% = 245/832 (max. 890 µg/kg) in Norway; 29.9% = 101/338 samples (max. 5865 µg/kg) in Finland; and 71.4% = 671/940 samples (max. 10,000 µg/kg) in the Netherlands. No NIV was found in the samples. The frequency of occurrence of DON in these wheat samples was positively correlated with temperature and relative humidity during the agricultural period. In contrast, levels of NIV found in samples of other cereals were negatively correlated with the above weather conditions. This indicates that some weather conditions may simultaneously facilitate biosynthesis of one mycotoxin and hamper biosynthesis of another [51]. Further research into the contamination of cereal grains with mycotoxins took place during the years 2007–2014 in Switzerland [52]. Findings showed that the frequency of occurrence and concentration in wheat of both DON and NIV depended on both agricultural season and region of cultivation (despite Switzerland’s relatively small geographical area). Similar dependence on region of cultivation was also shown in Japan by Yoshizawa et al. [53].

Numerous factors other than weather conditions may also influence the level of mycotoxin contamination of cereal grains. The genetic ability of a given fungi strain to biosynthesize toxins is among the most crucial of these factors [54,55]. It is noteworthy that some strains have been identified within the *Fusarium* family of fungi that are unable to biosynthesize mycotoxins [25,56]. Both *F. culmorum* and *F. graminearum* may biosynthesize DON and NIV, but a given strain does not synthesize both toxins. Champeil et al. [57] reported the coexistence of various fungi strains on the same plot of land. Regionalisation of various strains of *F. graminearum* is common, e.g., strains producing 3-AcDON in cereal grains are characteristic for Europe, China, Australia, and New Zealand, while strains producing 15-AcDON are more often found in the USA [54]. Other factors influencing the level of contamination of cereal grains with mycotoxins produced by the *Fusarium* fungi include the period for which cereal plants have been colonised by the fungi [26,27], the relationship between strain pathogenicity and wheat variety [58], the presence of fungicides (not every fungicide hampers biosynthesis of mycotoxins by fungi) [59,60], the presence of microflora antagonistic to *Fusarium* (capable of hampering pathogen development, e.g., *Trichoderma*) [61], the presence of agricultural crop residues (stubble remains), which may be a source of inoculum from previous agricultural seasons [30], and nitrogen fertilisation [62]. The sheer number of factors that may influence the development of FHB makes it difficult to show clearly the relationship between weather conditions and the level of mycotoxin contamination of wheat grain. Moreover, even the *Fusarium* fungi capable of bio-synthesizing mycotoxins do not necessarily produce trichothecenes [57].

The natural occurrence of NIV-3G in cereal grains has been studied by only a few authors. Yoshinari et al. [35] reported a 12–27% NIV-3G/NIV ratio in wheat grain, and quite a few reports on the natural occurrence of DON-3G in cereal grains have been published in recent years. The latter was always found on a concentration level lower than that of DON, although both levels were proportional. The reported DON-3G/DON ratios include 6–29% across various cereal varieties [63], 6–22% in durum wheat [64], less than 30% in durum wheat [65], 0–84% in wheat [66], 4–37% in wheat [38], and 2–48% across various cereal varieties [24]. The contemporary understanding is that major factors responsible for the response of wheat plants to an FHB infection, expressed as the DON-3G/DON ratio, include cultivation location and weather conditions, agricultural season, genotype, and mutual interaction between cultivation location and the genotype [66,67]. The resistance of wheat plants to FHB is directly proportional to their ability to convert DON into DON-3G [66,68]. Ovando-Martínez et al. [67] reported a rather strong correlation between DON and DON-3G (*R*^2^ = 0.872) in wheat artificially inoculated with *F. graminearum*. They also observed a systematically lower DON-3G/DON ratio in samples with higher DON concentrations (>30 mg/kg). Similar strong DON/DON-3G correlation was also reported by Dong et al. [66] and Amarasinghe et al. [68]. Considering that cereal plant enzymes are capable of metabolising NIV into NIV-3G just like DON into DON-3G (although reaction speed may be different for both reactions), it can be expected that the NIV-3G concentration depends on that of NIV in the same manner that the DON-3G concentration depends on that of DON. 

## 3. Conclusions

This work showed that cross-interaction of antibodies in immunoassay columns may be used to simultaneously determine DON, NIV, their glucosides, and 3-AcDON in wheat grain. It is one of the first reports, globally, on this and on the natural occurrence of NIV-3G in grain. The concentration of mycotoxins in the tested wheat samples was shown to depend on weather conditions prevailing during the wheat cultivation period, as these conditions may heavily influence development of the *Fusarium* fungi producing the mycotoxins. The warm and dry spring of 2018 in Poland was not favourable for *Fusarium* development, hence contamination of 2018 wheat samples was lower than contamination of 2017 samples. The maximum permissible DON level (specified in Commission Regulation 1881/2006) [19] was exceeded in 3 out of 150 samples harvested in 2017, but in none of our 2018 samples. DON/NIV glucosides, which very often accompany DON/NIV, may increase amount of DON and NIV. The lower DON concentration in wheat grain, the lower risk that the legally binding threshold is exceeded, even taking into account DON derivatives. Nevertheless, food safety consideration requires a wide-ranging discussion on the need to update legislation concerning the threshold in view of the fact that DON derivatives commonly accompany DON itself. Weather conditions are highly variable and a factor beyond any control. Tighter monitoring of mycotoxins in cereal grains as factors threating food safety seems to be necessary, especially in the context of global climate change.

## 4. Materials and Methods 

### 4.1. Reagents and Standards

Certified standards of NIV (100 µg/mL), DON (100 µg/mL), DON-3G (50 µg/kg), and 3/15-AcDON (100 µg/kg each) were purchased from Romer Labs (Tulln, Austria). The certified standard of NIV-3G (110 µg/mL) was isolated from wheat according to the procedure described by Yoshinari et al. [36]. Acetonitrile, methanol, and HPLC/LC-MS grade water were purchased from Witko (Łódź, Poland). DON-NIV WB immunoaffinity columns and PBS buffer solutions were purchased from Vicam (Watertown, MA, USA).

### 4.2. Research Material

**Figure 3:**
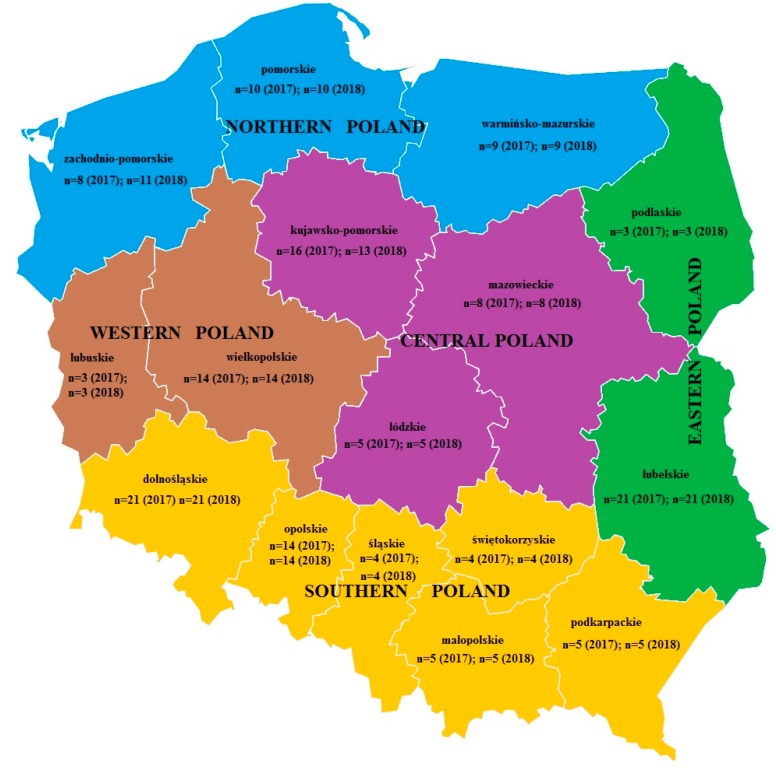
Breakdown of wheat sample quantities produced in 2017 and 2018 in 16 Poland’s voivodships, grouped into 5 regions.

To initiate the study, 300 samples of winter and spring wheat grain, each with a mass of approximately 1 kg, were sampled from crops harvested during two agricultural seasons, 2017 (*n* = 150) and 2018 (*n* = 150), and stored in various grain elevators all over Poland. As weather conditions in 2018 were unfavourable for cereal cultivation (low precipitation during the agricultural period, and an air temperature far from multi-year average), information on the origin of the samples was retained: *n* = 57, 34, 55, 48, and 106 samples originated from Northern, Western, Central, Eastern, and Southern Poland, respectively (Figure 3). Sampling was performed in accordance with guidelines specified in EC regulation 401/2006 of February 26, 2006 [34], which specifies appropriate methods of sampling and analysis for the official control of mycotoxin levels in foodstuffs. Next, each sample was ground in a Knife Mill Grindomix GM 200 (Retsch GmbH, Haan, Germany) lab grinder.

### 4.3. LC-MS

An H-class liquid chromatograph coupled with a mass spectrometer with a time-of-flight analyser (UPLC-TOF-HRMS, Waters, Milford, MA, USA) was used to determine mycotoxins. Analytes were separated on a 2.1 × 100 mm 1.6 µm UPLC C18 Cortecs chromatographic column (Waters, Milford, MA, USA) with an equivalent pre-column, operated in a gradient regime. Phase A was 90% methanol + 10% water, and phase B was 90% water + 10% methanol. Both phases contained 0.2% formic acid and 5 mM ammonium formate. The flow rate was 0.3 mL/min; the flow gradient was: 0–2 min 100% B; 3–6 min 50% B; 22–23 min 100% A, 25–28 min 100% B. Next, 5 µL volume samples were injected on the column. The mass spectrometer was operated in the positive and negative electrospray ionisation mode (ESI) and calibrated using leu-enkephalin solution. The ion source temperature was 150 °C, and the desolvation temperatures were 300 and 350 °C for positive and negative ionisation, respectively. The nebulizing gas (N_2_) flow rate was 750 L/min, and the cone gas flow rate was 40 L/min. Capillary bias was 3200 V. Ion optics worked in the V-mode. Ions used were as follows: *m*/*z* = 357.2 (M+FA-H)^−^ for NIV; *m*/*z* = 519.2 (M+FA-H)^−^ for NIV-3G; m/z = 341.2 (M+FA-H)^−^ for DON; *m*/*z* = 503.2 (M+FA-H)^−^ for DON-3G; and *m*/*z* = 339.2 (M+H)^+^ for 3-AcDON.

### 4.4. Sample Preparation 

**Figure 4:**
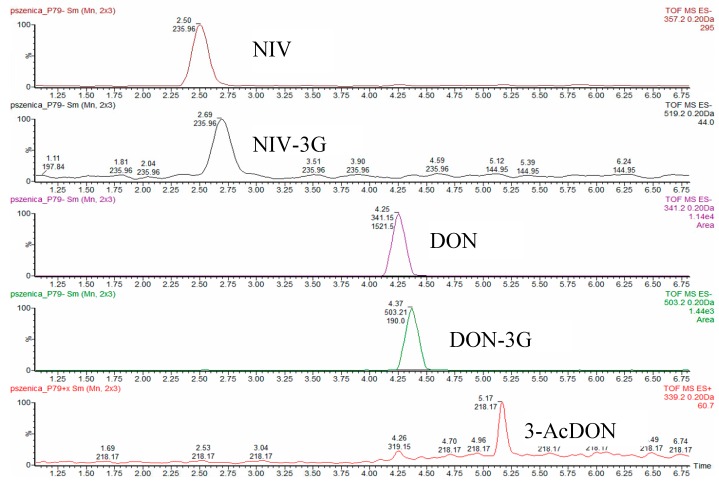
Typical UPLC-HRMS chromatograms of mycotoxins (NIV, NIV-3G, DON, DON-3G, and 3-AcDON) in naturally contaminated (real) wheat samples.

Both real and blank samples fortified with mix of standards (for method validation) were prepared in accordance with a slightly procedure described by Bryła et al. [38]. Briefly, 2 g of ground wheat grain put into a 50 mL falcon tube together with 8 mL of de-ionised water was first extracted in an Unidrive × 1000 homogenizer (CAT Scientific Inc., Paso Robles, CA, USA) for 2 min, then centrifuged in a MPV laboratory centrifuge (Med. Instruments, Warsaw, Poland) at 10,730× *g* for 10 min. Thereafter, 3 mL of the eluate was thinned with 3 mL of the PBS buffer, and 5 mL of the extract was purified on a DON-NIV WB immunoaffinity column (IAC). Next, the column was washed with 10 mL of PBS, and then with de-ionised water. Analytes, eluted first with 0.5 mL of methanol and then with 1.5 mL of acetonitrile, were collected into a reaction vial. Solvent was evaporated in a vacuum evaporator (45 °C), and residues were re-dissolved in 300 µL of 30% methanol solution. Typical chromatograms obtained from real wheat samples naturally contaminated with mycotoxins and purified on the IAC columns are shown in Figure 4.

### 4.5. Statistics

The experimental data were statistically evaluated using the Statgraphics 4.1 software package (Graphics Software System, STCC, Inc., Rockville, MD, USA). A one-way ANOVA was used to assess the significance of the differences between the determined mycotoxin concentrations. Fisher’s Least Significant Difference (LSD) test at α = 0.05 was used for the paired tests.

## Figures and Tables

**Table 1:** Recovery rates R% and method repeatability and precision (expressed as relative standard deviation RSD%) determined for four selected fortification levels.

Recovery (R) and Relative Standard Deviation (RSD)	NIV	NIV-3G	DON	DON-3G	3-AcDON	15-AcDON
fortification level for *n* = 4 (µg/kg)	88.5	52.9	96.2	9.7	97.8	97.8
R (%)	71.7	85.0	88.9	104.3	70.5	<10
RSD (%)	12.8	20.2	19.8	19.0	7.2	-
fortification level for *n* = 4 (µg/kg)	176.9	105.8	192.3	19.3	195.6	195.6
R (%)	77.6	83.5	98.0	88.0	71.8	<10
RSD (%)	8.9	9.4	12.5	13.2	7.3	-
fortification level for *n* = 4 (µg/kg)	265.4	158.6	288.5	29.0	293.4	293.4
R (%)	81.3	91.4	97.2	92.1	72.7	<10
RSD (%)	11.3	12.9	9.5	11.1	10.4	-
fortification level for *n* = 4 (µg/kg)	530.7	317.3	576.9	57.9	586.8	586.8
R (%)	78.3	82.7	99.9	84.5	69.5	<10
RSD (%)	5.7	4.5	7.8	12.1	7.3	-

**Table 2:** Average, median, min, and max concentration (μg/kg) of NIV, NIV-3G, DON, DON-3G, and 3-AcDON in wheat grain sampled in 2017 and 2018 in five different regions of Poland. NIV3G/NIV and DON3G/DON ratios are also shown. Letters denote groups in which average concentrations of mycotoxins in wheat during a given vegetation season were statistically different. Average values in total number of samples have been compared between both vegetation seasons.

Wheat Samples		Concentration (μg/kg)														
		NIV	NIV-3G	NIV-3G/NIV Molar Ratio		DON	DON-3G	DON-3G/DON Molar Ratio	3-AcDON	NIV	NIV-3G	NIV-3G/NIV Molar Ratio	DON	DON-3G	DON-3G/DON Molar Ratio	3-AcDON
		Season 2017								Season 2018						
Northern Poland *n* = 27 in 2017 *n* = 30 in 2018	Positive samples (%)	12 (44%)	5 (19%)		-	27 (100%)	21 (78%)	-	9 (33%)	12 (40%)	3 (10%)		15 (50%)	8 (27%)		4 (13%)
	Average	16.4 ^ab^	8.2 ^a^		20%	143.3 ^a^	37.5 ^a^	17%	3.2 ^a^	11.8 ^a^	5.7 ^a^	19%	11.4 ^a^	5.4 ^a^	23%	3.8 ^ab^
	Median	13.3	7.0		22%	75.5	30.1	15%	3.2	8.6	5.2	19%	9.5	4.7	25%	3.8
	Min–Max	8.4–45.0	5.0–14.7		20–36%	18.3–515.1	5.6–107.6	5–34%	2.3–4.0	8.0–22.5	5.0–6.8	18–20%	5.1–29.7	4.2–8.6	13–30%	3.0–4.4
Western Poland *n* = 17 in 2017 *n* = 17 in 2018	Positive samples (%)	3 (18%)	-		-	16 (94%)	13 (76%)	-	3 (18%)	10 (59%)	3 (18%)		6 (35%)	1 (6%)		1 (6%)
	Average	11.5 ^ab^	-		-	77.9 ^a^	18.6 ^ab^	14%	5.7 ^b^	14.5 ^ab^	5.9 ^a^	17%	7.8 ^ab^	4.7	34%	2.3
	Median	11.1	-		-	69.1	14.9	14%	5.8	11.1	5.2	20%	8.3	4.7	34%	2.3
	Min–Max	10.8–12.7	-		-	9.9–148.1	4.4–43.1	8–21%	2.5–8.9	8.0-26.6	5.0–7.4	12–20%	5.1–10.7	-	-	2.3
Central Poland *n* = 29 in 2017 *n* =26 in 2018	Positive samples (%)	17 (59%)	6 (21%)		-	28 (97%)	19 (66%)	-	10 (34%)	13 (50%)	4 (15%)		18 (69%)	9 (35%)		5 (19%)
	Average	19.1 ^ab^	8.8 ^a^		20%	164.5 ^a^	35.2 ^ab^	15%	3.6 ^a^	51.0 ^b^	16.5 ^a^	13%	15.5 ^ab^	5.6 ^a^	21%	6.5 ^a^
	Median	15.6	7.5		20%	81.5	16.5	13%	3.1	11.5	10.6	12%	9.2	4.4	21%	4.9
	Min–Max	8.1–73.7	6.2–13.5		12–25%	7.4–1260.9	4.4–150.3	3–34%	2.4–6.8	8.0–405.4	5.0-39.6	6–22%	5.3–57.1	4.0–10.2	7–54%	2.9–16.1
Eastern Poland *n* = 24 in 2017 *n* = 24 in 2018	Positive samples (%)	17 (71%)	4 (17%)		-	19 (79%)	9 (38%)	-	5 (21%)	19 (79%)	5 (21%)		16 (67%)	6 (25%)		2 (8%)
	Average	14.6 ^a^	7.2 ^a^		22%	111.4 ^a^	29.5 ^ab^	11%	2.7 ^a^	22.6 ^ab^	7.0 ^a^	15%	30.2 ^ab^	18.2 ^a^	24%	3.7 ^ab^
	Median	10.8	7.5		20%	18.5	6.2	11%	2.6	20.9	6.7	16%	7.6	5.7	21%	3.7
	Min–Max	8.1–34.3	6.0–7.9		15–35%	5.2–1670.7	4.0–217.2	6–19%	2.0-3.4	8.2–74.5	6.3–8.1	6–19%	5.0–303.1	4.0–82.1	10–40%	2.4–4.9
Southern Poland *n* = 53 in 2017 *n* = 53 in 2018	Positive samples (%)	24 (45%)	5 (9%)		-	28 (53%)	22 (42%)	-	6 (11%)	36 (68%)	5 (9%)		22 (42%)	17 (32%)	17 (32%)	7 (13%)
	Average	31.5 ^b^	10.7 ^a^		11%	91.7 ^a^	18.4 ^b^	14%	4.6 ^ab^	19.7 ^ab^	8.0 ^a^	15%	55.1 ^b^	15.1 ^a^	21%	3.3 ^b^
	Median	20.2	7.3		10%	39.5	10.4	14%	4.0	16.6	6.9	14%	13.7	4.9	21%	2.7
	Min–Max	8.5–185.6	5.5–26.8		7–14%	5.8–1428.7	4.0–199.6	7–27%	2.5–9.0	8.0–61.9	5.9–11.0	11–22%	5.0–461.7	4.0–87.7	10–31%	2.4
TOTAL	Positive samples (%)	73 (49%)	22 (15%)		-	138 (92%)	97 (65%)	-	38 (25%)	89 (59%)	20 (13%)		91 (61%)	50 (33%)		24 (16%)
	Average	21.4 ^a^	8.7 ^a^		19.5%	109.2 ^a^	25.8 ^a^	15%	3.6 ^a^	23.4 ^a^	8.8 ^a^	15.5%	32.4 ^b^	12.3 ^b^	21.9%	3.9 ^a^
	Median	15.0	7.4		20.0%	45.7	11.6	14%	3.2	14.6	6.7	15.8%	10.0	4.8	21.3%	3.0
	Min–Max	8.1–185.6	5.0–26.8		7–36%	5.2–1670.7	4.0–217.2	3–34%	2.0–9.0	8.0–405.4	5.0–39.6	6–22%	5.0–461.7	4.0–87.7	7–54%	2.2–16.1

Except for the TOTAL section, ^a,b^ denote statistically significant differences between average concentrations among various regions of the country within the given agricultural season; ^a,b^ in the TOTAL section denote statistically significant differences between the 2018/2017 agricultural seasons.

## Supplementary Materials

Figure S1: Distribution of temperature and precipitation in the growing seasons 2017 and 2018 in different regions of Poland. Data according to the metrology service of WeatherOnline Ltd. (WeatherOnline Ltd.—Meteorological Services, London, UK).
