# Supplementary material for: Contamination of Wheat Cultivated in Various Regions of Poland during 2017 and 2018 Agricultural Seasons with Selected Trichothecenes and Their Modified Forms
Source: Toxins (Basel). 2019 Feb 1;11(2):88. doi: 10.3390/toxins11020088 (PMC6409988; doi:10.3390/toxins11020088)
Supplement: Supplementary file 1 [file toxins-11-00088-s001.pdf]

toxins

Supplementary Materials: Contamination of Wheat Cultivated in Various Regions of Poland during 2017 and 2018 Agricultural Seasons with Selected Trichothecenes and Their Modified Forms

Marcin Bryła, Edyta Ksieniewicz-Woźniak, Tomoya Yoshinari, Agnieszka Waśkiewicz and Krystyna Szymczyk

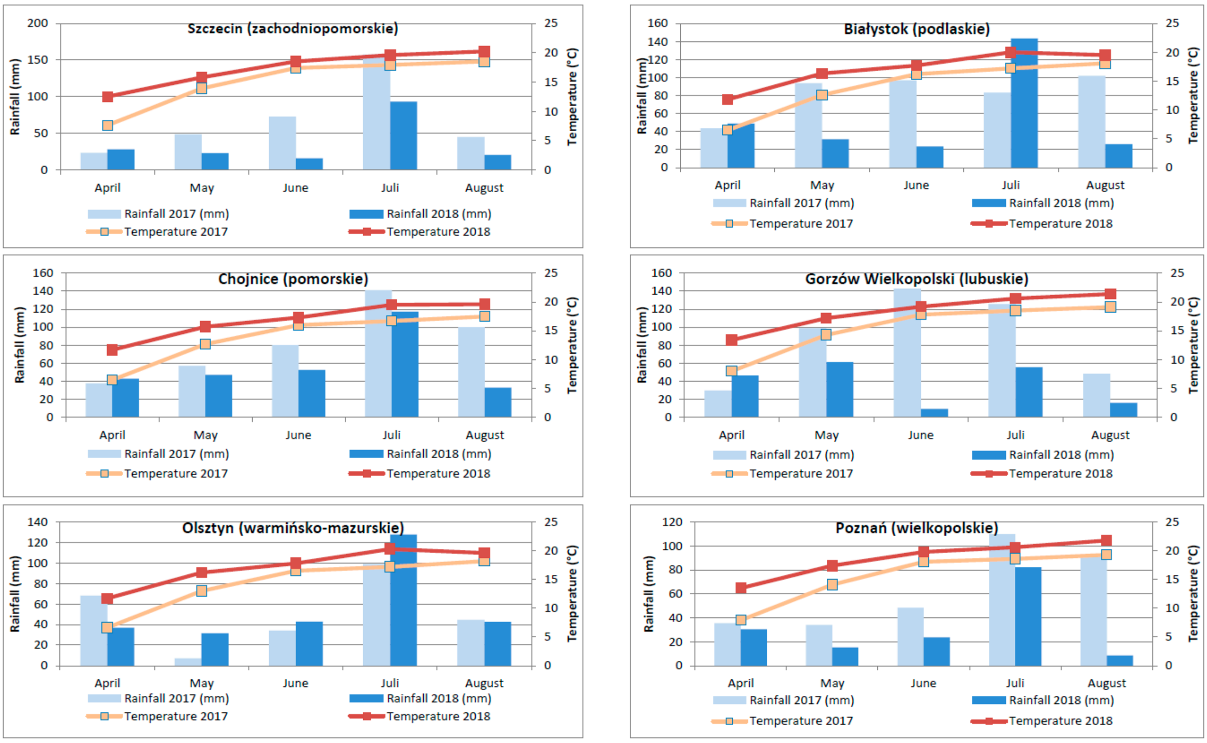

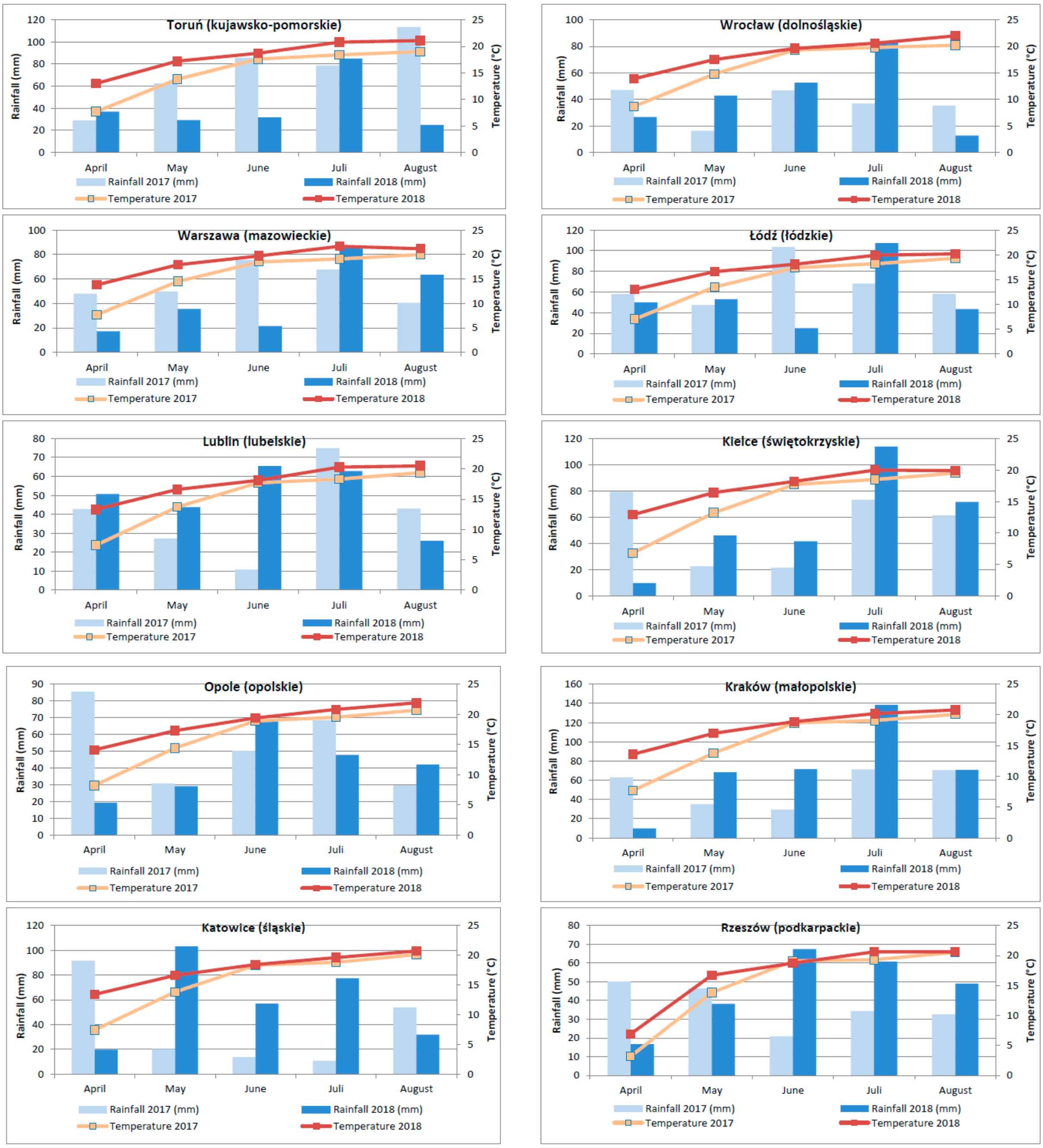

Figure 1. Distribution of temperature and precipitation in the growing seasons 2017 and 2018 in different regions of Poland. Data according to the metrology service of WeatherOnline Ltd. (WeatherOnline Ltd.—Meteorological Services, London, UK).
